# Acceptability, reach and implementation of a training to enhance teachers’ skills in physical activity promotion

**DOI:** 10.1186/s12889-020-09653-x

**Published:** 2020-10-16

**Authors:** Elina Renko, Keegan Knittle, Minttu Palsola, Taru Lintunen, Nelli Hankonen

**Affiliations:** 1 University of Helsinki, Social Psychology, Faculty of Social Sciences, P.O. Box Q3 54, Unioninkatu 37, 00014 Helsinki, Finland; 2 University of Jyväskylä, Sport and Exercise Psychology, Faculty of Sport and Health Sciences, Jyväskylä, Finland

**Keywords:** Acceptability, Implementation, teacher training, Theoretical framework of acceptability, Theory-based intervention, School-based interventions

## Abstract

**Background:**

To achieve real-world impacts, behavior change interventions need to be scaled up and broadly implemented. Implementation is challenging however, and the factors influencing successful implementation are not fully understood. This study describes the nationwide implementation of a complex theory-based program targeting physical activity and sedentary behavior in vocational schools (Lets’s Move It; LMI). The implementation primarily involved a systematic and theory-based training and user manual for school staff. We explore how the perceived acceptability of this training (in line with the Theoretical Framework of Acceptability) relates to (un) successful implementation. The study evaluates (1) the experienced acceptability of the training and anticipated acceptability of later delivering the program; (2) reach and implementation, including adaptations and barriers; (3) whether acceptability ratings predict teachers’ intentions for implementation.

**Methods:**

Upper secondary school staff from vocational and high schools (*n* = 194) enrolled in a two-part training, covering implementation of the LMI program and training in motivational interaction styles. One hundred fifty-one participants attended both parts of the training. Participants reported their perceived acceptability of the training and their implementation efforts in online questionnaires at baseline, after training sessions and at long-term follow-up. Qualitative data (open-ended questions) were analysed with content analysis to collate responses. Quantitative data analyses involved correlations and logistic regression.

**Results:**

Participants rated the training as highly acceptable on all dimensions (average ratings exceeded 4.0 on a 5-point scale). The implementation reached at least 6100 students and 341 school classes. Most teachers intended to continue program implementation. Acceptability ratings explained 51.7% of teachers’ intentions to implement the student program (훘^2^ = 30.08; df = 8; *p* < .001), with affective attitude, perceived effectiveness and self-efficacy the most influential. Teachers commonly reported condensing program content, and reported deficits of time and collegial support as common barriers to implementation.

**Conclusion:**

High acceptability and reach of the training indicate strong potential for implementation success. Multiple facets of acceptability seem important to successful implementation. Future research should explore ways to improve acceptability, thereby promoting successful implementation in real-world settings.

## Background

Given the insufficient levels of physical activity (PA) present among today’s youth [1], and the links between low levels of PA and the incidence of noncommunicable diseases [2], interventions are needed which can increase PA at population level. This might be particularly important among adolescents, as the transition into adulthood sets the stage for PA habits which carry into adult life [3]. As most adolescents spend a large proportion of their days in school, school-based interventions have been suggested to have high potential to create population-level improvements in PA [4]. Indeed, school-based PA interventions in children and adolescents have shown positive effects [5], and particularly among older adolescents have produced small-to-medium changes in PA [6].

Broad dissemination and implementation of effective evidence-based approaches is needed to adequately support PA in schools. At present though, there is a gap between what is demonstrated as efficacious in research settings and what is taken up by end-users in the real-world [7]. Implementation and dissemination of evidence-based interventions are emerging areas in PA research. Time is the most frequently reported factor influencing implementation of school PA interventions, followed by delivery agent efficacy, supportive school environment, technical support and training, and contextual appropriateness of interventions [8]. Further research has demonstrated the importance of teacher engagement in the maintenance of school-based programs [9, 10]. Taken together, it appears that numerous implementation-related contextual factors have an additive linear association with implementation [11].

For school-based PA interventions, it seems vital that teachers be equipped with the necessary skills and resources to overcome common barriers to implementation [10]. As conducting a school-based program to evoke and support sustainable behavior change extends beyond what is included in standard preservice teacher education, Continuing Professional Development (CPD) trainings could be key to helping teachers deliver interventions and improve instructional practices in this area [10, 12, 13]. Effective professional development teaches teachers and school staff new knowledge and skills that they can apply in practice, thereby promoting valued and sustainable organizational changes, including creating a school atmosphere that is conducive to PA participation [14, 15].

Despite the importance of teachers in implementation, CPD teacher training or in-service teacher training has received little attention within implementation research [10, 15]. A review from Lander et al. [10] found that the characteristics of teacher trainings used in school-based physical education (PE) were vastly under-reported, with only around one quarter of the included studies reporting on any aspect of the teacher training. Similarly, very few studies have investigated the extent to which teacher CPD trainings lead to the implementation of school-based PA promotion strategies in daily practice. The study we report here sheds light on this under-researched area, and focuses acutely on how the acceptability of CPD teacher trainings relates to real-world implementation [10, 15].

It is increasingly acknowledged that intervention acceptability should be considered in the development, evaluation and implementation of interventions, as it is a necessary precondition for an intervention to be successfully implemented and effective [16]. In line with the recently developed Theoretical Framework of Acceptability (TFA) [16], we define acceptability as a “multi-faceted construct that reflects the extent to which people delivering or receiving an intervention consider it to be appropriate, based on anticipated or experienced cognitive and emotional responses to the intervention”. The TFA proposes that acceptability consists of seven component constructs: attitude, burden, ethicality, intervention coherence, opportunity costs, perceived effectiveness and self-efficacy. These different dimensions allow for a fine-grained view of intervention acceptability. Acceptability can be measured before, during and after interventions, these are defined as prospective, concurrent and retrospective acceptability, respectively. Perhaps due to its novelty, no study to our knowledge has yet empirically evaluated intervention acceptability in line with the TFA. We present the first example in the context of an in-service teacher training for implementing a school-based PA program.

The in-service training in the focus of this study sought to give teachers and other school staff (e.g. classroom assistants and student counsellors) necessary skills to implement a school-based PA promotion intervention called Let’s Move It (LMI). Briefly described, the LMI intervention was designed to increase PA and decrease excessive sedentary behavior (SB) among vocational school students aged 18–22, especially those with low or moderate levels of PA. It was a multi-level intervention that targeted changes in both classroom and school environments, as well as targeting adolescents’ cognitions and motivations. LMI drew from self-determination theory (SDT) [17], self-regulation and planning theories [18, 19], habit theory [20], and utilized group motivational interviewing principles for the delivery [21, 22]. The intervention included a six-lesson program delivered to students to foster autonomous motivation and self-regulation skills for PA, as well as an additional booster session, and three workshops delivered to all teachers to teach them how to enable less sitting for their students in class. The intervention was delivered by project staff. The student program included individual and group activities to e.g. enrich adolescents’ understanding of PA, help find personally important reasons to increase PA, to foster motivation to try out different types of PA to find an activity they enjoy (or want to learn to enjoy), and then use a set of self-regulation skills to regularly engage in PA in their leisure-time. For more details on the development and content of the LMI, see [23].

The LMI intervention was evaluated in a cluster-randomised controlled trial (RCT) [24], where the student program and teacher workshops were delivered by the research team. As the intervention led to meaningful improvements in levels of light PA and was well received by targets, (Hankonen, Haukkala, Palsola, Heino, Sund, Tokola, et al. Effectiveness of the Let’s Move It multilevel school-based intervention on physical activity and sedentary behavior: A cluster randomized trial, submitted.) efforts were made to begin disseminating the LMI intervention more broadly, as research-based interventions need to be scaled up in order to have practical impact in the real world. However, as it would not have been feasible to have members of the research team deliver the LMI intervention as part of a broader roll out, a training was developed to offer teachers and other school staff the needed skills to implement core components of the LMI intervention in practice in their own schools.

In line with existing best-practice evidence [10], the developed in-service training was theory-based (the scientific background of LMI see p. 6), offered comprehensive subject and pedagogical content (e.g. how to use LMI materials and different interaction techniques), and consisted of two parts delivered on separate days. The training focused on offering participants the skills and materials necessary to support their students’ autonomous motivation for PA, and to provide workshops to other teachers on how to reduce student sitting and utilize motivational interaction techniques.

The current study focuses on acceptability, reach and implementation of this theory- and evidence based teacher training. As acceptability of an intervention is a necessary precursor for implementation [16], this study also investigates the relationship between acceptability and later implementation in practice and explores which acceptability dimensions best predict intentions for implementation. The above presented definition of acceptability [16] provides a hypothesis according to which “cognitive and emotional responses are likely to influence behavioural engagement with the intervention”. The current study will be the first one to utilize the recently developed TFA to test whether prospective (i.e. anticipated) and retrospective (i.e. experienced) acceptability ratings are related to later implementation. To clarify, experienced acceptability is about the acceptability of the implementation strategy, and the anticipated acceptability is about the perceived acceptability of the LMI (see the research question 1 below).

More specifically, this study will examine three sets of investigations: (1) the *experienced* acceptability of participating in teacher training Parts I and II, and the *anticipated* acceptability of later delivering the trained programs (i.e. the LMI student program, and the LMI teacher workshops); (2) the extent to which teacher training participants (intend to) implement trained programs in their own schools following the training, the adaptations they make when implementing the program, and factors that will facilitate or hinder their implementation; and (3) the extent to which acceptability ratings were related to subsequent (intentions for) implementation of the trained programs.

All questions were pre-registered on the Open Science Framework (OSF) before accessing the data (https://osf.io/cnzfb/).

## Methods

In this longitudinal field implementation study, data were collected at four measurement points over the course of 3 to 4 months. The study protocol received a favourable review from the University of Helsinki Ethical Review Board in the Humanities and Social and Behavioural Sciences (Statement 26/2017).

### Recruitment and participants

The newsletters of our collaborators and stakeholders (e.g. Association of Physical and Health Educators of Finland) were employed to recruit participants for a in-service teacher training in upper secondary schools. Both general and vocational upper secondary education were recruited to participate. Participants were also recruited through social media (e.g., announcements shared to Facebook pages or groups that the target group teachers typically follow) and events organized by collaborators and stakeholders of the project. Ultimately, 17 trainings were organized during the school year 2017–2018, and they took place in different regions of Finland. Participants of the training were school staff, mostly teachers (92%) from 30 different upper secondary schools (both general and vocational upper secondary education), 65% from vocational and 35% from high schools. On average, they had 15.4 years of working experience (ranging from 0 to 38). 59.9% of participants were female and the average age was 44.5 (ranging from 17 to 64).

### Training

The teacher training aimed to enhance teachers’ skills in physical activity promotion. All trainings were delivered by a project coordinator, who had substantial experience in piloting and delivering workshops and trainings about the topic. She delivered the training either face-to-face (*n* = 13) or online (*n* = 4 as a video conference via Adobe Connect). Both trainings had the same content. Schools or teachers did not receive any incentives for participating. All trainings were delivered during working hours and free of charge. They took place during the school year 2017–2018 (both during the autumn and spring semester).

The teacher training consisted of two parts which were carried out on separate days. The average length of time in between two training sessions was 2 weeks. Each part involved a standardized 4-h group training. The training was previously developed in close collaboration with upper secondary school teachers and collaborators of LMI. The training content is described briefly below and in more detail in the separate file (see Additional file 1).

#### Part I: program delivery training dedicated to the content of the LMI intervention

Through interactive exercises and discussions, participants got introduced to the materials of LMI intervention and learned how to use them to provide *the LMI program to their students* as well as *the LMI sitting reduction workshops to teacher colleagues* in the future.

#### Part II: motivational interaction training

Through practical examples, interactive exercises and discussions, participants got acquainted with SDT as the theoretical framework, concepts of motivation, basic psychological needs (i.e. competence, relatedness and autonomy), and practical interaction techniques to foster self-determined motivation. These included (1) Understanding resistance and using non-controlling language (e.g. avoiding words such as “must” or “have to”), (2) providing choices (e.g. letting students choose between two alternative activities for the lesson, instead of teacher deciding), (3) advising without pressing, (4) empathising and reflective listening, (5) positive feedback and appreciation, (6) open questions and interest, and (7) providing structure and rationale (e.g. explaining what will happen on the lesson, and providing justifications for activities).

### Data collection

**Fig. 1:**
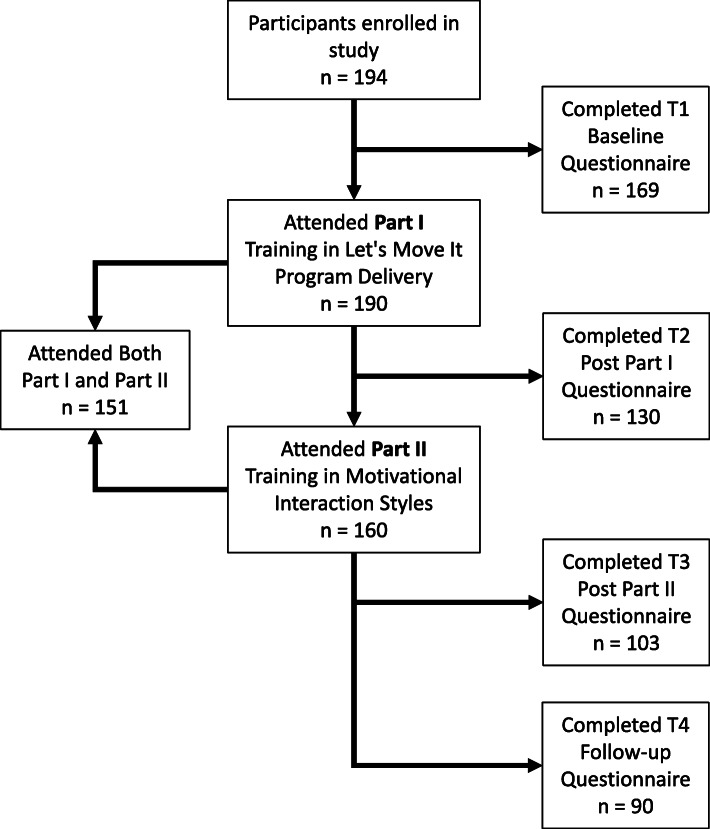
Flow of participants

One hundred ninety-four participants enrolled in study. One hundred ninety participants attended the Part I (Program delivery) and one hundred sixty participants attended the Part II (Motivational interaction). One hundred fifty-one participants attended both parts of the training. Participants (see Fig. 1) were asked to complete four short online questionnaires. The questionnaire was developed for this study and has been published on the OSF (https://osf.io/e84ga/). First, approximately 1 week before the Part I, project coordinator invited all participants via email to complete the baseline questionnaire online. The first questionnaire included items about motivational style (i.e. Time 1, T1 baseline), these results will be reported elsewhere (author et al., in prep.).

Then, approximately 1 day after each training part, the project coordinator invited all participants by email to complete an online questionnaire to obtain data on their beliefs concerning the experienced acceptability of the training, and the anticipated acceptability of applying the trained programs (T2 after Part I) and the proposed interaction techniques (T3 after Part II) in their own work (i.e. immediate post-training). The T3 questionnaire also included items about intentions to use the interaction techniques. On four occasions, the project coordinator invited the participants to complete the questionnaire immediately after the training (of these, in three cases the questionnaire was completed online and due to technical difficulties, the project coordinator had to allow the participants to complete the questionnaire for one group of participants as a paper-version questionnaire).

Approximately 2 to 3 months after the Part II, participants were sent an email invitation to complete an online questionnaire (T4) on their intentions to apply and actual implementation of trained programs and the proposed interaction techniques. The questionnaire also included items about motivational style. Email reminders were sent to participants who had not filled this questionnaire by May 2018.

All participants who responded to questionnaires concerning training acceptability (T2 & T3) attended that specific part of the training and all participants who responded to follow-up questionnaire (T4) attended at least one part of the training.

### Measures

In the baseline questionnaire, respondents reported their gender and other background factors, including occupation: PE/HE/both, other teachers, other staff. All respondents were included in the analysis concerning the research questions 1 & 2. However, “other staff” were excluded from the analyses concerning the research question 3 (the extent to which acceptability ratings were related to subsequent (intentions for) implementation of the trained programs). The later three questionnaires assessed acceptability (T2 & T3), reach and implementation (T4) of the training.

#### Acceptability

Acceptability items (T2 & T3) were created in accordance with the Theoretical Framework of Acceptability (TFA) [14] so that they would closely reflect the theoretical definition of each acceptability facet (Affective attitude, Burden, Ethicality, Intervention coherence, Opportunity costs, Perceived effectiveness, Self-efficacy) in the context of these particular interventions. In line with TFA [14], we measured both *experienced acceptability* of the training, as well as the *anticipated acceptability* of the programs that the participants will later deliver in their schools (i.e., student program and teacher workshops). Responses to these items ranged on a scale from 1 “strongly disagree” to 5 “strongly agree”. Additional file 2 shows an overview of acceptability items.

#### Reach, implementation and intentions to implement

To assess reach (T4), participants were asked to provide open-ended numbers to the questions, “Please estimate how many student groups your implementation of the LMI measures reached in total” and “Please estimate the amount of teachers who participated in sitting reduction workshops you have delivered”.

To assess implementation and intended implementation of interventions (T4), we used two questions: “To what extent have you implemented the LMI student program and do you intend to implement it in future?” and “To what extent have you implemented the teacher sedentary behavior reduction workshops and do you intend to implement it in future?” Response options for both questions were: “Whole program” or “Partly”.

Based on responses to these questions, we classified the extents to which participants had implemented the program (i.e. had not implemented any part of the program, or had implemented at least part of the program), and the extents to which they intended to implement the program in future (i.e. planned to implement the full program, planned to implement part of the program, or did not plan to implement any of the program). This resulted in the categories shown in Table 2.

#### Adaptations

To assess adaptations in delivery (T4), we used the following open-ended questions: “Which parts have you delivered or what kind of changes have you made in the student program?” and “Which parts have you delivered, or what kind of changes have you made in sedentary behavior reduction workshops?” In the analyses of the LMI components delivered by the participants, we investigated what number of different types of components the participants described delivering, and then counted how many reports fall under each component. Responses describing similar components were categorized under one overarching type (e.g., ‘I have taken breaks to do 1-2 minute exercises’ and ‘I have used activity breaks’ both fall under ‘Activity breaks in classes’).

#### Perceived barriers

To assess perceived barriers (T4), we used the following item: “If you have not delivered student program and/or sedentary behavior reduction workshops for teachers, why have not you? Feel free to choose multiple response options.” This was followed by 11 typical barriers and an open field for other reasons.

### Analyses

Pearson correlations examined the associations between measures of experienced and anticipated acceptability (T2 & T3). Open-ended questions regarding program implementation were analysed with content analysis (T4): Responses were inductively coded. The process of inductive coding meant that the data was coded without trying to fit it into a pre-existing coding frame [25]. Initial codes were generated by MP (an excel file was used to list the codes but no specific code book was developed) and collated into potential categories by MP and ER. The focus of collating codes was on mentions of different implementation methods. The categories were reviewed with co-authors and original quotes were left visible for the author group to validate the categories. There were no disagreements about the categories. Logistic regression analyses, controlled for type of teacher (i.e. PE/health education (HE)/both, other teachers, other staff), investigated the extents to which acceptability ratings of the training sessions (T2 & T3) predicted teachers intentions to subsequently implement the teacher workshops and student program (T4). All statistical analyses were conducted using SPSS 24.0.

## Results

### Acceptability of interventions among training participants

Teacher training was well-accepted. Participants reported high experienced and anticipated acceptability scores (Table 1 & Additional file 3).

**Table 1:** Means (SDs) and correlations (item-level) in Experienced and Anticipated Acceptability

	N	Mean	Std. Deviation	α	1	2	3	4
1. Experienced acceptability, Part I	129	4,48	0,47	.640		,594^a^	,521^a^	,205
2. Anticipated acceptability, Student sessions, Part I	128	4,22	0,55	.744			,587^a^	,236^b^
3. Anticipated acceptability, Teacher workshops, Part I	127	4,12	0,60	.759				,281^b^
4. Experienced acceptability, Part II	103	4,44	0,49	.566				

The mean acceptability scores presented here represent the means of all assessed acceptability dimensions assessed for a given type of acceptability. Further detail on this, as well as the scores for each separate acceptability dimension, are presented in Additional file 3

^a^.Correlation is significant at the 0.01 level (2-tailed)

^b^.Correlation is significant at the 0.05 level (2-tailed)

#### What was the experienced acceptability of the training?

In evaluating Part I (Program delivery training) in the role of training participant, participants reported high experienced acceptability scores on the dimensions of affective attitude, burden (reversed), perceived effectiveness and intervention coherence. The proportion of participants who gave a rating of the two highest scores (on a scale from 1 “strongly disagree” to 5 “strongly agree”) was 98% for affective attitude, 91% for burden (reversed), 88 and 87% for perceived effectiveness and 98% for intervention coherence.

Similarly, results demonstrated high experienced acceptability scores of the training Part II (Motivational interaction training) on the dimensions of affective attitude, burden (reversed), intervention coherence, perceived effectiveness and self-efficacy. The proportion of participants who gave a rating of the two highest scores (on a scale from 1 “strongly disagree” to 5 “strongly agree”) was 97% for affective attitude, 85% for burden (reversed), 98% for intervention coherence, 92% for perceived effectiveness and 83% for self-efficacy.

#### What was the anticipated acceptability of later delivering the programs?

In evaluating how they feel about delivering *the student program* to their students in the future (in the role of the provider), participants reported on average high anticipated acceptability scores on the dimensions of affective attitude, burden (reversed), intervention coherence and perceived effectiveness. Self-efficacy obtained slightly lower scores. The proportion of participants who gave a rating of the two highest scores (on a scale from 1 “strongly disagree” to 5 “strongly agree”) was 91% for affective attitude, 81% for burden (reversed), 87% for intervention coherence, 93% for perceived effectiveness and 67% for self-efficacy.

Similarly, participants reported high anticipated acceptability scores for delivering *the teacher workshops* to teacher colleagues in the future. Results showed high scores on all anticipated acceptability measures, self-efficacy obtained slightly lower scores. The proportion of participants who gave a rating of the two highest scores (on a scale from 1 “strongly disagree” to 5 “strongly agree”) was 79% for affective attitude, 81% for burden (reversed), 90% for intervention coherence, 79% for perceived effectiveness and 72% for self-efficacy.

Compared to delivering the student program, affective attitude and perceived effectiveness was rated slightly lower for the teacher workshops.

Finally, participants were asked how they feel about using *the interaction techniques* they have learned during the training with their students in the future. Results showed high anticipated acceptability scores on the dimensions of affective attitude, burden (reversed), ethicality, perceived effectiveness and self-efficacy. The proportion of participants who gave a rating of the two highest scores (on a scale from 1 “strongly disagree” to 5 “strongly agree”) was 96% for affective attitude, 88% for burden (reversed), 86% for ethicality, 95% for perceived effectiveness and 94% for self-efficacy (Additional file 4).

In terms of the anticipated opportunity costs for using the interaction techniques or practicing their use, mostly participants did not anticipate any trouble or problems, or that they would have to give up something else important. A few participants pointed out *limited time as an anticipated problem*.

Regarding opportunity costs for implementing student program or teacher workshops, mostly participants did not anticipate trouble or problems, nor that they would have to give up something else important. The most commonly mentioned problems were related to *time and resources*. Besides, participants pointed out that it could be hard to teach colleagues and convince them of the importance of the topic. These opportunity costs may explain why the anticipated self-efficacy scores of delivering the student program (Mean 3.91) and teacher workshops (Mean 3.98) were lower than anticipated self-efficacy of using the interaction techniques (Mean 4.38).

### Implementation and reach of the trained programs in schools

The second set of research questions focused on implementation (i.e. participants’ delivery of both student program and teacher workshops). At follow-up T4, the estimated implementation reach of the student program was 6118 students and 341 student groups in total (*n* = 48). Majority (74.5%) of the participants reported having either fully or partly implemented the student program and all intended to continue with it fully or partly, whereas 22.2% of the participants had not yet implemented the program but intended to start. 3.3% of participants had not implemented nor intended to implement the program (Table 2).

**Table 2:** The extent to which the participants have (intended) to implement the interventions

	Intention for full (and partial) implementation	Intention for only partial implementation	No intention for implementation	In total
**STUDENT INTERVENTION (*n*** **= 91)**				
Has implemented fully	7 (7.7%)	1 (1.1%)	0 (0.0%)	8 (8.8%)
Has implemented partly	17 (18.7%)	42 (46.2%)	0 (0.0%)	59 (64.9%)
Has not implemented	13 (14.3%)	8 (8.8%)	3 (3.3%)	23 (65.4%)
In total	37 (40.7%)	51 (56.1%)	3 (3.3%)	91 (100%)
**TEACHER INTERVENTION / SEDENTARY BEHAVIOUR REDUCTION PROGRAM (*n*** **= 83)**				
Has implemented fully	9 (10.8%)	0 (0.0%)	0 (0.0%)	9 (10.8%)
Has implemented partly	6 (7.2%)	21 (25.3%)	3 (3.6%)	30 (36.1%)
Has not implemented	21 (25.3%)	14 (16.9%)	9 (10.8%)	44 (53.0%)
In total	36 (43.4%)	35 (42.2%)	12 (14.5%)	83 (100%)

As for the teacher workshops, 46.3% participants reported having either fully or partly implemented workshops for their colleagues and 42.7% intended to continue with it, whereas 53.7% participants reported having not yet implemented the workshops but 46.3% intended to start. A small minority (3.7%) of the participants reported having either fully or partly implemented the workshops and do not intend to continue with it, and 10.9% reported having not implemented nor intended to implement the workshops. (Table 2).

#### What adaptations were made when implementing the programs?

In the open-ended questions about adaptations, six respondents gave clear statements of delivering *the student program*, and 2 respondents reported having not delivered any parts. Most responses were about the SB reduction activities undertaken by the participants themselves rather than about the program delivery to the students. Hence, it was not clear whether or not the participants actually delivered the lessons or just adopted the measures they report. Three respondents reported making delivery adaptations by having a tighter schedule, condensing the content, and creating an online course.

The most popular intervention activities included activity breaks, asking students to get up to collect materials and going outside during the school day, all of which are in scope of LMI. Many of the respondents reported reducing SB and increasing PA in their classes but did not specify the means. Respondents also reported having spread the word about the program in their organization.

Eleven respondents clearly reported having delivered *the teacher workshops* in the open-ended questions. The most common adaptations were to combine the material into one-time session or otherwise condense the content (Additional file 5). While not having reported delivering the program, the respondents reported having spread the word of the program, shared tips, presented the program to their colleagues, and shared program materials. A handful of respondents reported increasing activity in their meetings and reducing SB in their own work (Additional file 5).

#### What facilitated or hindered implementation?

The most commonly reported reasons for not implementing the program were related to the *lack of time, (26.4%)* or to the *need for more collegial support (5.1%)* (Table 3). The content analysis of other reasons (8.6%) revealed that some of the schools were currently taking part in a *similar program* and are hence already implemented similar ideas. Moreover, additional barriers for implementation were related to *lack of opportunities within work roles*: participants currently had no classes to teach or otherwise felt that they were not teaching suitable classes for program delivery. None selected inadequate materials, perceiving program or workshop delivery unimportant, ineffective program or workshops or believing that students would not like the LMI exercises as barriers. Twenty-eight respondents (14%) reported receiving additional funding (helping in the intervention implementation).

**Table 3:** Types of implementation & perceived barriers for implementation

Types of LMI student program implementations (***n*** = 69)	Number of reports on this type of implementation	% of all reported types
Activity breaks in class	33	35.9
SB reduction (not specified)	11	12.0
Students are asked to get up and collect materials from teachers’ desk	10	10.9
Going outside with students during classes	7	7.6
Spreading the word in the organization	5	5.4
Organizing lessons in a way that promotes physical activity	5	5.4
Stretching during classes	4	4.3
External activities or events for the students	3	3.3
Learning Café teaching style for in-class work	2	2.2
Physically active ways of voting e.g. by standing up	2	2.2
Using the posters for activation	2	2.2
Non-specific	8	8.7
**In total**	**92**	**100**
**Perceived barriers for implementation of both programmes (*n*** **= 52)**	**Frequency**	**% of 197**
Other work tasks take too much time	35	17.8
Hard to find time for teacher workshops	17	8.6
Need for more collegial support for program or workshop delivery	10	5.1
No organizational support	2	1
Need for more monetary resources to deliver desired parts (e.g. to buy equipment)	2	1
I feel that I could not deliver the program or workshops well enough	2	1
I believe that teachers do not like the workshops	1	0.5
Inadequate materials	0	0
Program or workshop delivery is not important	0	0
Ineffective program or workshops	0	0
Students would not like the LMI exercises	0	0
Other reasons	17	8.6
**In total**	**86**	**43.6**

### The extent to which acceptability ratings predict intentions to implement the trained programs

A logistic regression analysis controlled for job role (i.e. PE teacher, HE teacher or other classroom teacher) found that acceptability ratings explained 51.7% of the variance in teachers’ intentions to implement the student program (Chi-Square = 30.08; df = 8; *p* < .001). Of the acceptability dimensions included in the model, affective attitude (Wald = 7.43; *p* = .006), perceived effectiveness (Wald = 6.41; *p* = .011) and self-efficacy (Wald = 5.04; *p* = .025) predicted teachers’ intentions to implement the student program (see Table 4). Neither intervention coherence nor burden (reversed) predicted intentions (all nonsignificant). The model correctly predicted the presence of intentions to implement the student program for 80.6% of the included cases.

**Table 4:** Logistic regression of teachers’ intentions to implement the student program (*N* = 62)

		B	S.E.	Wald	df	Sig.	Exp(B)
Model 1	Type of school^a^						
	Teaching at a vocational school	0.329	0.542	0.369	1	0.544	1.390
	Job role						
	PE and/or HE teacher (reference)			0.024	2	0.988	
	Not a teacher	21.468	23,205.422	0.000	1	0.999	2,106,592,942.627
	Other teacher	−0.083	0.536	0.024	1	0.877	0.921
	*R*^*2*^ *= 12.1%*						
Model 2	Type of school						
	Teaching at a vocational school	−0.525	0.758	0.480	1	0.488	0.592
	Type of teacher						
	PE and/or HE teacher (reference)			2.271	2	0.321	
	Not a teacher	22.718	22,097.283	0.000	1	0.999	7,351,348,455.463
	Other teacher	1.222	0.811	2.271	1	0.132	3.394
	Affective attitude	2.882	1.057	7.427	1	0.006	17.846
	Intervention coherence	−1.361	0.858	2.519	1	0.112	0.256
	Perceived effectiveness	−2.318	0.916	6.406	1	0.011	0.098
	Self-efficacy	1.634	0.728	5.042	1	0.025	5.126
	Burden (reversed)	−0.336	0.583	0.333	1	0.564	0.714
	*R*^*2*^ *= 51.7%*						

^*a*^ Reference category for type of school: Teaching at a upper secondary schoolModel 1 *Nagelkerke R*^2^ = 12.1%; Model 2 *Nagelkerke R*^2^ = 51.7%

When considering implementation of the teacher workshops, a logistic regression analysis controlled for job role indicated that acceptability ratings of the training explained 29.8% of the variance in teachers’ intentions to implement (Chi-Square = 17.15; df = 7; *p* = .016) (see Table 5). Of the dimensions of acceptability included in the model, only self-efficacy predicted intentions to implement the teacher program (Wald = 4.56; *p* = .033). The model correctly predicted the presence of intentions to implement the teacher workshops for 76.5% of the included cases.

**Table 5:** Logistic regression of teachers’ intentions to implement the teacher workshops (*N* = 68)

		B	S.E.	Wald	df	Sig.	Exp(B)
Model 1	Job role						
	PE and/or HE teacher (reference)			0.313	2	0.855	
	Not a teacher	0.325	0.795	0.167	1	0.682	1.385
	Other teacher	0.256	0.520	0.243	1	0.622	1.292
	*Nagelkerke R*^*2*^ *= 0.6%*						
Model 2	Type of teacher						
	PE and/or HE teacher (reference)			2.924	2	0.232	
	Not a teacher	1.008	1.044	0.932	1	0.334	2.741
	Other teacher	1.211	0.717	2.851	1	0.091	3.357
	Affective attitude	−0.368	0.566	0.424	1	0.515	0.692
	Intervention coherence	0.947	0.579	2.670	1	0.102	2.577
	Perceived effectiveness	0.221	0.512	0.186	1	0.666	1.248
	Self-efficacy	1.223	0.573	4.558	1	0.033	3.398
	Burden (reversed)	−0.461	0.369	1.566	1	0.211	0.630
	*Nagelkerke R*^*2*^ *= 29.8%*						

## Discussion

This study investigated the acceptability, reach and implementation of the in-service training to implement the LMI intervention across Finnish upper secondary schools. Our findings revealed high experienced and anticipated acceptability scores for the training. Furthermore, the estimate of students reached via trained participants was wide, and levels of later implementation, as well as intentions for implementation, were also high. Finally, several dimensions of acceptability were related to intentions to subsequently implement the program.

Previous research highlights the importance of teacher engagement for successful implementation of school-based programs [9, 10]. Acceptability of the program should be a key consideration when designing, implementing and evaluating it [16]. In this study school staff reported high acceptability scores both as training participants (the experienced acceptability of the training) and as intervention providers (the anticipated acceptability of the programs that the participants will later deliver in their schools).

In line with TFA’s assumption that acceptability - cognitive and emotional responses - may impact behavioural engagement with the intervention [16], we found that acceptability ratings indeed were related to subsequent implementation of the program. Of the assessed dimensions of acceptability, affective attitude, perceived effectiveness and self-efficacy predicted teachers’ intentions to implement the student program, whereas only self-efficacy was related to subsequent intentions to implement the teacher workshops. More specifically, those rating the student program to be more enjoyable to deliver, perceiving it to be effective in promoting student PA and feeling self-efficacious to deliver the program, were more likely to intend to implement the program in the future. Those feeling self-efficacious to deliver the teacher workshops were more likely to intend to continue. Our findings are in agreement with a systematic review [8], finding that self-efficacy (e.g., ease of implementation, teacher’s skill proficiency) and perceived benefits of innovation (cf. perceived effectiveness) were among the prominent categories that influence implementation of school PA interventions. It should be noted that as some variables had high skew, not all variables were able to present a proper test of the relationship, i.e. some null findings may be due to low variance.

These findings highlight the multi-faceted definition of the acceptability construct, and possible differences in their predictive value. More research is needed to find out what role each dimension of acceptability plays in predicting implementation, and decisions about which facets of acceptability to investigate should be guided by research aims. For example, a feasibility study prior to a definitive trial would benefit from measures to estimate whether the minimum threshold of acceptability can be reached, without the consideration of potential for normally distributed responses. On the contrary, a study testing the relationships to other variables, using traditional multivariate methods, may want to construct scales in a way that the required normal distribution assumptions can be fulfilled and an adequate test presented.

Evidence-based strategies that target multiple implementation contextual factors need to be developed to optimally support schools to implement PA interventions [11]. However, the overall quality of evidence on how effective strategies are is low and lacks consistent terminology. Thus, it is often difficult to assess whether strategies are cost-effective and improve the implementation of school-based policies or practices [26]. In this study, the most common perceived implementation barriers were lack of time and the need for more collegial support. These results align with previous findings according to which time constraints, supportive organizational environment and resource availability/quality were the most prevalent factors that influenced implementation of school PA interventions in most implementation studies [8]. In our study, time and resource -related troubles were not only the most common perceived barriers for implementation, but also the most commonly mentioned problems that implementing the student program or teacher workshops would *cause* (i.e. opportunity costs). Further, supportive organizational environment was considered to be an important opportunity cost since participants pointed out that it could be hard to convince colleagues of the importance of the topic. The reported implementation of the student program was higher than implementation of the teacher workshops and it might be that the most frequently endorsed barriers to implementation (time, resources and supportive organizational environment) are more pressing when the audience consists of colleagues. In sum, the barriers for implementation were unrelated to the program itself, and were more external, organisational factors [27]. On the other hand, one could argue that the program itself should include components to more efficiently help get collegial support and be less resource-intensive. Although complex and multi-component interventions have been suggested to be more effective, the complexity is a double-edged sword due to being a barrier, requiring time and potentially challenging provider self-efficacy. We did not utilize any specific framework for assessing implementation and adaptations, e.g., FRAME [28] as such frameworks were not available at the time of writing this paper. Using such framework might give more insight into for example the reasons for adaptations and the issues they may cause in terms of fidelity. Here, for instance, condensing content could be seen as a reactive adaptation to existing time constraints. Findings like these could help similar interventions to guide efforts to proactive modification earlier in the process and so lessen the risk for e.g., fidelity issues.

In the future, to improve (intentions for) implementation of teacher-delivered interventions, we recommend assessing anticipated acceptability multidimensionally early on. Unsurprisingly, our results would encourage that teacher trainings focus on fostering self-efficacy, which could happen by providing actual skills, proper demonstrations, opportunities to practice delivery in role-play simulations, to name just a few examples. Threats to self-efficacy may be different for programs delivered to students and to teacher colleagues, and qualitative formative research could best help optimise acceptability of interventions.

This study has several strengths. First, it sheds light on an under-researched and under-reported areas of both teachers’ role in school-based intervention implementation [8], and fine-grained empirical studies of intervention acceptability. To our knowledge, this study was the first one to utilize the TFA to test relationships between anticipated and experienced acceptability ratings and later implementation. We were able to test these previously untested relationships in real-life settings. Besides, this study identified the barriers and opportunity costs of implementation. Strategies that target these barriers/opportunity costs may improve later implementation of programs in the school setting. We also focus attention on the issues of nature and quality of teacher training and representing a comprehensive description of its characteristics (see the supplementary file: Training content), hoping to contribute to future studies. Clearer and more consistent reporting of teacher training characteristics has been called for to better inform the design of future interventions [10, 15]. Finally, the pre-registration of the research questions prior to accessing the data increases transparency.

This study also has some limitations. First, as in all self-report measures, there are limitations in participants’ willingness and ability to accurately report the later implementation and reach of the intervention. Additionally, we did no measurement of students’ PA, which may be particularly important in light of recent meta-analyses suggest lack of effects of school-based interventions on accelerometry-measured outcomes [29]. Second, regarding intentions for implementation it is important to recognize the discrepancy between individuals’ reports of good intentions to engage in behaviour and failure to act on those intentions (so called intention-behaviour gap). Third, despite a relatively large sample, we should be careful in generalizing the results. Additional research is warranted to better understand on what extent do the dimensions of rated acceptability predict intentions to implement interventions in different context. Fourth, the approach to implementation to schools was limited to teacher trainings mostly, and was therefore suboptimal. Improved impacts would have been gained by more intensive engagement of school management, intensive support for implementation, school champions, and a longer follow-up period. However, these were not realistic considering the project resource constraints, and on the other hand, the organisational and structural support was provided simultaneously by our partner organisation, the Finnish Schools On The Move. Finally, it should also be noted that as in any longitudinal study, validity can be threatened by dropout.

## Conclusions

To conclude, the results revealed high acceptability of the evaluated in-service training, and the trained programs. The level of estimated later implementation was high, the estimated reach was wide and several acceptability dimensions predicted teachers’ intentions to implement the programs. In particular, liking the program, perceiving it to be effective, and feeling self-efficacious to deliver it, were predictive of intentions to further implement it. Effective implementation may benefit from ensuring provider acceptability on many dimensions. The results demonstrate the importance of assessing both the experienced and the anticipated acceptability and shed light on the under-researched relationship between acceptability and implementation.

## Supplementary information

**Additional file 1.** The in-service professional development training (2 × 4 h) for teachers to implement the Let’s Move It program with a motivational interaction style: Description of the training content.

**Additional file 2.** Acceptability items.

**Additional file 3.** Experienced acceptability (EA) and anticipated acceptability (AA).

**Additional file 4.** Anticipated acceptability (AA), Interaction techniques, Part II.

**Additional file 5.** Types of adaptations and implementations in the teacher workshop delivery by those respondents who report having delivered or intending to deliver the program in some form.

## Data Availability

The datasets used and/or analysed during the current study are available from the corresponding author on reasonable request.

## Abbreviations

HE: health education
LMI: Let’s Move It (a complex theory-based program to promote physical activity and reduce sedentary behavior in vocational schools)
OSF: the Open Science Framework
PA: physical activity
PE: physical education
RCT: cluster-randomised controlled trial
SB: sedentary behavior
SDT: self-determination theory
TFA: the Theoretical Framework of Acceptability
