# Supplementary material for: Acceptability, reach and implementation of a training to enhance teachers’ skills in physical activity promotion
Source: BMC Public Health. 2020 Oct 16;20:1568. doi: 10.1186/s12889-020-09653-x (PMC7574409; doi:10.1186/s12889-020-09653-x)
Supplement: Supplementary file 1 — Additional file 1. The in-service professional development training (2 × 4 h) for teachers to implement the Let’s Move It program with a motivational interaction style: Description of the training content. [file 12889_2020_9653_MOESM1_ESM.docx]

**The in-service professional development training (2 x 4h) for teachers to implement the Let’s Move It program with a motivational interaction style: Description of the training content
Elisa Kaaja, Elina Renko, Nelli Hankonen (2018-2019)**

The training is divided into two half-day workshops, delivered on separate days (e.g. two weeks between the workshops). Participation in both workshops is recommended, but it is possible to subscribe only for one of them. Workshop 1 is dedicated to the content of the Let’s Move It program (materials and how to use them). The training starts with an introduction to behavior change, where we also briefly go through how interaction is connected to motivation.

Workshop 2 is dedicated to the topic of need-supportive/motivational interaction. The training starts with a theory part where the different types of motivation and the basic psychological needs as defined by Self-Determination Theory (SDT; Deci & Ryan, 2000; Ryan & Deci, 2017) are discussed. We also present practical examples of teaching practices that either foster students’ autonomous motivation and how teachers can avoid controlling interaction styles. After that, we go through seven practical teaching strategies that are useful to foster students’ autonomous motivation, thereby providing opportunities for discussion, hands-on practices and active learning during the session.

**Objectives:**

**Training Part I: Program Delivery Training**

After Part I, participants…

- understand the scientific background of Let’s Move It (LMI) program
- understand the basic model of behaviour change and how the LMI program has been developed
- understand the importance of physical activation for their students (i.e. learning, concentration, well-being)
- are able to utilize LMI materials in their own schools (both students’ and teachers’ materials)
- are aware of pragmatic tips on how to implement sitting reduction strategies in class

**Training Part II: Motivational Interaction Training**

After Part II, participants…

- understand the core principles of SDT (i.e. types of motivation and psychological needs)
- know how to use different interaction techniques that support people’s basic psychological needs for autonomy, competence and relatedness and thereby foster autonomous motivation
- are able to use need-supportive teaching strategies when they encounter unmotivated and low active student or teacher colleague
- know how to create a positive and peaceful atmosphere in the classroom

|  | **Part I: Program Delivery Training (approx. 4 hours)** |  |
| --- | --- | --- |
| **Exercise** | **Activity description** | **Materials** |
| **Pre-assignment:** | Fill in the SIS -questionnaire (Aelterman et al., 2018) |  |
| **Welcome**  **(15 min)** | Aims, assessment etc. | Ppt slides |
| **Mini-lecture:** Principles of behavior change and Let’s Move It material  **(15 min)** | The workshop facilitator gives a brief overview of main influences on behavior (change), motivation, and the LMI program.  - What do we do after motivation is enhanced?  - How to bridge the motivation-behavior gap? | Ppt slides |
| **Warming up exercise:** Motivating vs. non-motivating interaction (role play & discussion)  **(15 min)** | The facilitator explains that this role play exercise contains two different interaction styles. This exercise is adapted from an example in MI (Miller WR, Rollnick S. Motivational Interviewing – Helping People Change. 3rd ed. Guilford Press; 2012. 482 p. 6.). In pairs, the participants play out the teacher and student roles, and then discuss how they felt in these roles. Pairs talk about enhancing PA by using both styles (pressure, judgement, controlling language, closed question vs. open ended questions, providing choice, positive feedback, encourage change talk). After a few minutes, the facilitator asks how participants felt after these different role play discussions. The facilitator uses questions such as: *What was the role of the target person? How was the relationship between the adviser and the target person? How personalised advice did the target person likely perceive to have received in both examples?* | Ppt slides |
| **Teacher program:** Mini-lecture, familiarization with the teacher workshop program materials, LMI Teacher Workshop 1  **(70 min)** | Mini-lecture (appr. 10-15 minutes) on why sitting reduction workshops are important in schools (i.e. demonstrating this by showing participants some of the teacher feedback for LMI RCT) and how participants can arrange teacher workshops in their own schools in practice.  The facilitator distributes LMI teacher workshop materials to the participants.  The facilitator delivers the first LMI sitting reduction workshop: Participants take part in the first LMI sitting reduction workshop in order to gain personal experience on how the workshop is ideally delivered. In this workshop they  - are asked about the consequences of uninterrupted sitting (*“What kind of consequences do you know / have you experienced after prolonged sitting?”*). After a short group discussion, the facilitator makes a brief oral summary of it and shows a concise list of evidence-based consequences of uninterrupted sitting (a Let’s Move It poster displayed on the screen), in order to help participants get insight of the variety of benefits of sitting reduction.  - reflect on why students’ sitting reduction is or would be personally important to them. This is done in a group exercise in which the facilitator asks everyone to choose a personally meaningful reason to reduce sitting in classrooms (adapted from a similar exercise Hankonen et al. 2017). Participants pick up a card showing a personally meaningful reason. Next, in a circle they share their reason to the group, along with more elaboration, if they want.  - try out different ways to reduce students sitting in a Learning Café exercise in which four checkpoints are placed in different corners of the room. Participants circulate in small groups from check point to another, where written guidance is given to complete the check point task. At each check point, the small groups discuss questions concerning sitting reduction, with the aim of familiarising themselves with concrete tips how to reduce sitting and create more active classroom environments. These concrete tips (e.g. “Opinion Poll Queue”, “Voting with your Body”, use of equipment for light physical activity in different situations, forming groups in a physically active way, activity break posters and videos) are explained in detail in the teachers’ workbook. During this Learning Café exercise, participants also learn practical pedagogical ways to motivate students to participate in sitting breaks (e.g., how to give positive feedback, how to avoid coercion, how to ask students’ opinion, etc.).  (For more information on the teacher workshop content, see Köykkä et al., 2019, JESP). | Ppt slides  Video material (LMI website  and activity breaks)  LMI teachers’ workbook for students’ sitting reduction (printed)  LMI teacher program slides (printed)  Quick start guide to sitting reduction (printed)  Cards showing benefits of sitting reduction in class  Learning Café posters (printed)  LMI Posters (printed) |
| **Group discussions**: Challenging interaction situations with teacher colleagues  **(20 min)** | Facilitator shows challenging interaction situation scenarios (e.g. “*If one of your colleagues says that sitting reduction is totally pointless and only disturbs teaching, how could you react in a need supportive way without dismissing your colleague’s thought*s?”). Participants also have an opportunity to bring situations from their own experience to the general discussion. After a few minutes of small group discussions, participants share their solutions to the entire group, and the facilitator gives few tips on how to handle such situations, and gives examples how such tips were successfully handled by the research group during the field study. | Ppt slides |
| **Student program**  **(50 min)** | Mini-lecture (appr. 15-20 minutes) on the structure and content of the LMI program targeting students (6 sessions in total), including the most important messages. The messages are consolidated into six LMI principles, to make it clear and transparent what our underlying ideas are and what possible misconceptions we are targeting.   - **ADDING ANY MOVEMENT IS GOOD! ANY ACTIVITY IS BETTER THAN NOTHING –** Targeting the misconception that physical activity should be “all or nothing”, and that taking baby steps (“graded tasks”) is useful. Building sense of competence and self-efficacy. - **YOUR OWN CHOICE: WHETHER YOU ARE ACTIVE, AND HOW –** Emphasizing that it is everyone’s personal choice whether or not they are active, and the LMI does not force anyone for more PA. Supporting the need of autonomy. - **WE ARE ALL ENTITLED TO ACTIVITY** – Emphasizing opportunities; emphasizing that you do not need to be of a certain body shape or size to enjoy PA. - **KNOW WHAT MOVES YOU –** Emphasizing that it is important to identify one’s key personal motivation for PA. LMI explicitly supports formation of internal goals (in line with goal content theory of the SDT). - **GOAL: WELL-BEING, NOT FATLESS BODY** – Targeting the usual misconception that PA is mostly about getting lean and good-looking. These goal motivations are not explicitly endorsed by the LMI due to their potential inefficacy and side-effects, and instead, more intrinsic goals are suggested. - **SITTING SUCKS** – Informing the students in a non-moralizing way that sitting is a health risk independent of levels of PA and providing rationale why activity is important.   (More information on the program is available in Hankonen et al., 2016 and Hankonen et al., pre-print at <https://psyarxiv.com/ak68f>)  The facilitator distributes the LMI student program materials. The manual covers six 45-60-minute sessions that the teachers can deliver to students within health education and PE courses.  First, the facilitator shows some of the key exercises and explains their learning objectives and how they support the basic psychological needs. Then (s)he explains that due to time constraints, not all of the sessions can be taught in this workshop in detail and that therefore the participants will now have time to silently familiarise themselves with the first part of the manual, followed by supportive questions and discussion.  Participants familiarise themselves with the first part of the manual, focusing on the first session (appr. 5-10 minutes). Next, teacher participants answer a few questions about the first session, with the aim of checking what the teacher participants have understood based on reading the manual only. The questions included e.g. *“Which exercise in the first session aimed to help students identify personally important and meaningful reasons to be physically active?”*. After reading the manual, the teachers were given an opportunity to go through the questions either alone or with another participant. | Ppt slides  LMI student program slides and materials (printed)  LMI student program manual for teachers (printed) |
| **Summary & Feeling cards excercise**  **(5 min)** | Participants pick up a postcard with a picture that describes their current feeling. In this exercise, they have the opportunity to share their feelings and concerns about the LMI student program based on what they have just read from the manual, heard from the facilitator, and discussed with the group.  In case any critical or negative feedback arises, the facilitator tries to understand the position of the participants, acknowledging participants’ perspectives, problems and feelings. The key is to receive the negative feedback and show acceptance towards the person being critical, not to start to defending or explaining. The facilitator may say something along the lines of “*It’s important to raise concerns, and some parts are bound to seem at least a bit strange to many of you. Are there any other things that some of you are pondering?*” and when all criticism has come out and is accepted “*Maybe by giving the program a chance you will feel differently during the next workshop when we go more into practical ways to support the discussion in the classroom*”.  The facilitator can also acknowledge that he/she knows that there are a lot of new materials and information in this program and it is perfectly understandable and normal that it is difficult or challenging to grasp all of the content at once. He/she may also encourage to keep on familiarizing oneself with the materials later on, and ask more questions if needed. | Printed “feeling cards” |

|  | **Part II: Motivational Interaction Training (approx. 4 hours)** |  |
| --- | --- | --- |
| **Exercise** | **Activity description** | **Materials** |
| **Welcome**  **(5 min)** | Aims, assessment etc. | **Welcome:** Ppt slides |
| **Review of the previous workshop and progress**  **(15 min)** | Reviewing the first workshop, discussing thoughts and feelings raised by the first workshop, as well as observations triggered by what they learnt. What have participants done during past two weeks in terms of the previous session’s content (e.g. sitting reduction experiments, workshop organization)? (general discussion) | Ppt slides |
| **Mini-lecture and discussions** about different types of motivation  **(30 min)** | Minilecture.  Worksheet is modified from “*Verschillende types motivatie*” sheet (see Aelterman et al., 2013 in TATE, and Aelterman et al., 2014 in JSEP) | Ppt slides  Different types of motivation -sheets (SDT) and pens |
| **Small group discussions**  **(30 min)** | Real-life example about a need-based approach  The facilitator displays a slide of a dialogue between Minna “The teacher” and Mikko “The student” on the wall. In this dialogue Mikko declines to participate in a gymnastic exercise and the teacher starts to engage in a very controlling motivation style. The facilitator asks the participants to take the perspective of the student. How would student Mikko feel in this interaction with his teacher? How are Mikko’s basic psychological needs being supported/thwarted? And how might Minna, the teacher, feel after this kind of dialogue?  (small group discussion)  After a whole group discussion, the facilitator asks participants to come up with alternative, more need-supportive ways to handle the situation. | Ppt slides  Real-life example, a translation of “*Dialogue between coach Mikael and the 11-year old Marie”* (see Aelterman et al., 2013 in TATE, and Aelterman et al., 2014 in JSEP |
| **Intrinsic and extrinsic PA goals**  (**10 min)** | A short introduction to different quality (intrinsic vs. extrinsic) PA goals & discussion with activating style. | Ppt slides |
| **Need-supportive teaching strategies** to foster autonomous motivation  **(90 min)** | Theory, followed by discussions and role play/demo video examples:    First, the facilitator briefly presents all seven motivational interaction techniques or skills (need-supportive teaching strategies).  Motivational interaction techniques/skills:  (1) a style understanding resistance and non-controlling language  (2) empathizing and reflective listening,  (3) open questions & interest,  (4) advising without pressing,  (5) positive feedback & appreciation,  (6) providing choice,  (7) providing structure and rationale  Then, the facilitator asks if participants would like to concentrate on some specific techniques (= providing choice, soliciting participants’ input). This in an example of congruent teaching (‘teach as you preach’), where the trainers adopt a need-supportive approach themselves. If no wishes arise, the whole group will go through all the 7 techniques.  Facilitator distributes self-monitoring sheets (with a list of the techniques and a possibility to log use of them) and Brief guide of Motivational interaction. In this guide, the main principles of the SDT and the content of Training Part II are being explained. Also, some practical examples (in PE classes) of motivating and controlling style are listed. | Ppt slides  Video material (advising with and without pressing; change talk examples)  Printed: Brief guide on Motivational interaction  Self-monitoring sheets (printed) |
| **Voluntary Homework** | Self-monitoring of using techniques in daily life:  -Choose 1-3 motivational interaction techniques and keep a diary about the experiences of using them. |  |
| **Summary & Feeling Cards exercise**  **(5 min)** | Reviewing participants’ experiences, see Training Part I last exercise.  Facilitator may ask each participant to tell *“The most important thing for me here today was…”*, but also other prompts for group discussion can be used to wrap up the session and recap the most important insights of the workshops. | Printed “feeling cards” |

**Project: “Enhancing physical activity promotion in upper secondary schools: Nationwide dissemination and implementation of evidence-based intervention strategies”**

**Principal Investigator: Nelli Hankonen, University of Helsinki, Finland**

**Funder:** Academy of Finland, Forging Ahead With Research, 2016-18.

**Funding number**: 304114

**References:**

Aelterman, N., Vansteenkiste, M., Van Keer, H., De Meyer, J., Van den Berghe, L., & Haerens, L. (2013). Development and evaluation of a training on need-supportive teaching in physical education: Qualitative and quantitative findings. *Teaching and Teacher Education, 29*, 64–75. <https://doi.org/10.1016/j.tate.2012.09.001>

Aelterman, N., Vansteenkiste, M., Van den Berghe, L., De Meyer, J., & Haerens, L. (2014). Fostering a Need-Supportive Teaching Style: Intervention Effects on Physical Education Teachers’ Beliefs and Teaching Behaviors. *Journal of Sport and Exercise Psychology, 36*(6), 595–609. <https://doi.org/10.1123/jsep.2013-0229>

Deci, E. L., & Ryan, R. M. (2000). The ‘What’ and ‘Why’ of Goal Pursuits: Human Needs and the Self-Determination of Behavior. *Psychological Inquiry, 11(*4), 227–268. https://doi.org/10.1207/S15327965PLI1104_01

Hankonen, N., Heino, M. T. J., Araújo-Soares, V., Sniehotta, F. F., Sund, R., Vasankari, T., … Haukkala, A. (2016). ‘Let’s Move It’ – a school-based multilevel intervention to increase physical activity and reduce sedentary behaviour among older adolescents in vocational secondary schools: a study protocol for a cluster-randomised trial. *BMC Public Health, 16*(1), 451. <https://doi.org/10.1186/s12889-016-3094-x>

Hankonen, N., Absetz, P., & Araújo-Soares, V. (2019). Changing activity behaviors in vocational school students: The stepwise development of the ‘Let’s Move it’ intervention. *Under review.* https://doi.org/10.31234/osf.io/ak68f

Köykkä, K., Absetz, P., Araújo-Soares, V., Knittle, K., Sniehotta, F. F., & Hankonen, N. (2019). Combining the reasoned action approach and habit formation to reduce sitting time in classrooms: Outcome and process evaluation of the Let’s Move It teacher intervention. *Journal of Experimental Social Psychology, 81*, 27–38. https://doi.org/10.1016/j.jesp.2018.08.004

Miller, W. R., & Rollnick, S. (2012). *Motivational Interviewing: Helping People Change*. New York, NY: Guilford Press.

Ryan, R. M., & Deci, E. L. (2017). *Self-Determination Theory: Basic Psychological Needs in Motivation, Development, and Wellness.* New York: Guilford Publications.
