# Supplementary material for: Acceptability, reach and implementation of a training to enhance teachers’ skills in physical activity promotion
Source: BMC Public Health. 2020 Oct 16;20:1568. doi: 10.1186/s12889-020-09653-x (PMC7574409; doi:10.1186/s12889-020-09653-x)
Supplement: Supplementary file 2 — Additional file 2. Acceptability items. [file 12889_2020_9653_MOESM2_ESM.docx]

**Additional file 2. Acceptability items.**

| **Component construct** | **Theoretical definition** | **Items measuring experienced acceptability of training (as participants)** | | **Items measuring anticipated acceptability of delivering (as providers)** | | |
| --- | --- | --- | --- | --- | --- | --- |
| (Sekhon et al. 2017) | | **Part I** | **Part II** | **Part I** | **Part I** | **Part II** |
|  |  | **Program delivery training** | **Interaction training** | **Delivering LMI student sessions** | **Delivering workshops for teachers** | **Using interaction techniques** |
| **Affective attitude** | How an individual feels about the intervention | I enjoyed attending the training | I enjoyed attending the training | I would enjoy it | I would enjoy it | I would enjoy it |
| **Burden** | The perceived amount of effort that is required to participate in the intervention | Attending the training required too much effort | Attending the training required too much effort | It would require too much effort | It would require too much effort | It would require too much effort |
| **Ethicality** | The extent to which the intervention has good fit with an individual’s value | N/A | N/A | N/A | N/A | It would not be ethical or in line with my personal values |
| **Intervention coherence** | The extent to which the participant understands the intervention, how it addresses their condition and how it works | Content of the training was easy to understand and follow | Content of the training was easy to understand and follow | Delivering the Let’s Move It student sessions is a comprehensible and appropriate task | Delivering the Let’s Move It teacher workshops is a comprehensible and appropriate task | N/A |
| **Opportunity costs** | The extent to which benefits, profits or values that must be given up to engage in the intervention | N/A | N/A | Do you think implementing the Let’s Move It program student sessions in your school would cause you trouble or problems, or you would have to give up something else important because of it? | Do you think implementing the Let’s Move It program teacher workshops in your school would cause you trouble or problems, or you would have to give up something else important because of it? | Do you think using the interaction techniques or practicing their use would cause you trouble or problems, or you would have to give up something else important because of it? |
| **Perceived effectiveness** | The extent to which the intervention is perceived as likely to achieve its purpose | The training offered me sufficient tools to use the Let’s Move It materials and principles in my school with students (to promote PA) / with my teacher colleagues (to deliver sitting reduction workshops | The training provided me an understanding of the processes of motivational interaction | I think the Let’s Move It student sessions would be effective in promoting physical activity among students | I believe that the workshops would make my colleagues decrease students’ excessive sitting in their classrooms | It would be effective in motivating students |
|  |  |  | I learned concrete techniques that I can use in everyday interaction |  |  |  |
| **Self-efficacy** | The participant’s confidence that they can perform the behaviour(s) required to participate in the intervention | N/A | N/A | I am certain I would be able to deliver the student sessions as intended | I am certain I would be able to deliver the workshops as intended | I believe I would be able to use the interaction skills as intended |
