# Supplementary material for: Acceptability, reach and implementation of a training to enhance teachers’ skills in physical activity promotion
Source: BMC Public Health. 2020 Oct 16;20:1568. doi: 10.1186/s12889-020-09653-x (PMC7574409; doi:10.1186/s12889-020-09653-x)
Supplement: Supplementary file 3 — Additional file 3. Experienced acceptability (EA) and anticipated acceptability (AA). [file 12889_2020_9653_MOESM3_ESM.docx]

**Additional file 3.** **Experienced acceptability (EA) and anticipated acceptability (AA).**

|  | **n** | **Mean** | **SD** | **α** | **2** | **3** | **4** | **5** | **6** | **7** | **8** | **9** | **10** | **11** | **12** | **13** | **14** | **15** | **16** | **17** | **18** | **19** | **20** | **21** | **22** | **23** | **24** | **25** | **26** |
| --- | --- | --- | --- | --- | --- | --- | --- | --- | --- | --- | --- | --- | --- | --- | --- | --- | --- | --- | --- | --- | --- | --- | --- | --- | --- | --- | --- | --- | --- |
| **1. Experienced acceptability Part I (EA 1), sum** |  | 4,48 | 0,47 | .640 | ,64^**^ | ,57^**^ | ,51^**^ | ,81^**^ | ,73^**^ | ,58^**^ | ,59^**^ | ,40^**^ | ,41^**^ | ,53^**^ | ,41^**^ | ,39^**^ | ,52^**^ | ,51^**^ | ,29^**^ | ,49^**^ | ,21^*^ | ,39^**^ | ,21 | ,29^**^ | ,02 | ,19 | ,17 | ,17 | ,12 |
| 2. EA 1 affective attitude | 118 | 4,60 | 0,54 |  |  | ,19^*^ | ,25^**^ | ,46^**^ | ,37^**^ | ,51^**^ | ,43^**^ | ,44^**^ | ,26^**^ | ,36^**^ | ,26^**^ | ,24^**^ | ,35^**^ | ,37^**^ | ,21^*^ | ,39^**^ | ,10 | ,22^*^ | ,37^**^ | ,35^**^ | ,31^**^ | ,31^**^ | ,17 | ,22 | ,27^*^ |
| 3. EA 1 burden (reversed) | 128 | 4,54 | 0,97 |  |  |  | ,09 | ,17 | ,046 | ,20^*^ | ,28^**^ | ,06 | ,31^**^ | ,13 | ,36^**^ | ,13 | ,17 | ,20^*^ | ,20^*^ | ,09 | ,08 | ,05 | -,03 | 0,13 | -,09 | -,01 | -,01 | -,05 | -,02 |
| 4. EA 1 intervention coherence | 127 | 4,73 | 0,48 |  |  |  |  | ,32^**^ | ,27^**^ | ,18^*^ | ,32^**^ | ,17 | ,19^*^ | ,54^**^ | ,22^*^ | ,09 | ,24^**^ | ,21^*^ | ,16 | ,32^**^ | ,02 | ,17 | ,18 | ,01 | ,05 | ,32^**^ | ,24^*^ | ,05 | -,04 |
| 5. EA 1 perceived effectiveness (PA) | 129 | 4,31 | 0,74 |  |  |  |  |  | ,78^**^ | ,60^**^ | ,55^**^ | ,42^**^ | ,35^**^ | ,47^**^ | ,23^*^ | ,46^**^ | ,44^**^ | ,40^**^ | ,21^*^ | ,38^**^ | ,18^*^ | ,42^**^ | ,10 | ,20 | -,05 | ,09 | ,11 | ,21 | ,11 |
| 6. EA 1 perceived effectiveness (SB) | 128 | 4,26 | 0,76 |  |  |  |  |  |  | ,47^**^ | ,43^**^ | ,31^**^ | ,20^*^ | ,43^**^ | ,22^*^ | ,37^**^ | ,57^**^ | ,54^**^ | ,22^*^ | ,55^**^ | ,29^**^ | ,50^**^ | ,21 | ,32^**^ | ,04 | ,12 | ,16 | ,22 | ,20 |
| 7. EA 1 recommendation | 128 | 4,55 | 0,70 |  |  |  |  |  |  |  | ,46^**^ | ,31^**^ | ,29^**^ | ,31^**^ | ,25^**^ | ,44^**^ | ,52^**^ | ,47^**^ | ,24^**^ | ,42^**^ | ,29^**^ | ,46^**^ | ,26^*^ | ,29^**^ | ,18 | ,22 | ,07 | ,18 | ,25^*^ |
| **8. Anticipated acceptability, Student sessions, Part I (AA S1), sum** |  | 4,22 | 0,55 | .744 |  |  |  |  |  |  |  | ,73^**^ | ,71^**^ | ,71^**^ | ,58^**^ | ,77^**^ | ,59^**^ | ,42^**^ | ,40^**^ | ,53^**^ | ,26^**^ | ,50^**^ | ,24^*^ | ,19 | ,14 | ,24^*^ | ,15 | ,15 | ,21 |
| 9. AA S1 affective attitude | 128 | 4,34 | 0,73 |  |  |  |  |  |  |  |  |  | ,41^**^ | ,42^**^ | ,28^**^ | ,49^**^ | ,28^**^ | ,26^**^ | ,17 | ,33^**^ | ,06 | ,22^*^ | ,09 | 0,12 | ,13 | ,03 | -,02 | ,07 | ,08 |
| 10. AA S1 burden (reversed) | 127 | 4,24 | 0,91 |  |  |  |  |  |  |  |  |  |  | ,33^**^ | ,24^**^ | ,41^**^ | ,42^**^ | ,27^**^ | ,51^**^ | ,31^**^ | ,12 | ,25^**^ | ,12 | 0,06 | ,04 | ,14 | ,10 | ,12 | ,21 |
| 11. AA S1 intervention coherence | 127 | 4,32 | 0,70 |  |  |  |  |  |  |  |  |  |  |  | ,42^**^ | ,42^**^ | ,52^**^ | ,38^**^ | ,20^*^ | ,66^**^ | ,26^**^ | ,41^**^ | ,35^**^ | ,32^**^ | ,11 | ,38^**^ | ,30^**^ | ,23^*^ | ,17 |
| 12. AA S1 perceived effectiveness | 127 | 4,28 | 0,62 |  |  |  |  |  |  |  |  |  |  |  |  | ,29^**^ | ,38^**^ | ,25^**^ | ,21^*^ | ,37^**^ | ,36^**^ | ,22^*^ | ,22 | ,11 | ,093 | ,19 | ,22^*^ | ,13 | ,21 |
| 13. AE S1 self-efficacy | 128 | 3,91 | 0,94 |  |  |  |  |  |  |  |  |  |  |  |  |  | ,48^**^ | ,32^**^ | ,27^**^ | ,29^**^ | ,17 | ,63^**^ | ,12 | 0,11 | ,17 | ,15 | -,01 | ,03 | ,077 |
| **14. Anticipated acceptability, Teacher workshops, Part I (AA T1), sum** |  | 4,12 | 0,60 | .759 |  |  |  |  |  |  |  |  |  |  |  |  |  | ,82^**^ | ,65^**^ | ,70^**^ | ,64^**^ | ,77^**^ | ,28^*^ | ,33^**^ | ,065 | ,22^*^ | ,25^*^ | ,25^*^ | ,31^**^ |
| 15. AA T1 affective attitude | 127 | 4,09 | 0,83 |  |  |  |  |  |  |  |  |  |  |  |  |  |  |  | ,44^**^ | ,50^**^ | ,41^**^ | ,59^**^ | ,20 | ,30^**^ | -,07 | ,15 | ,22 | ,25^*^ | ,27^*^ |
| 16. AA T1 burden (reversed) | 125 | 4,14 | 0,95 |  |  |  |  |  |  |  |  |  |  |  |  |  |  |  |  | ,21^*^ | ,22^*^ | ,34^**^ | ,16 | ,12 | ,03 | ,16 | ,22 | ,12 | ,16 |
| 17. AA T1 intervention coherence | 125 | 4,34 | 0,70 |  |  |  |  |  |  |  |  |  |  |  |  |  |  |  |  |  | ,44^**^ | ,47^**^ | ,33^**^ | ,37^**^ | ,15 | ,34^**^ | ,18 | ,21 | ,34^**^ |
| 18. AA T1 perceived effectiveness | 127 | 4,05 | 0,78 |  |  |  |  |  |  |  |  |  |  |  |  |  |  |  |  |  |  | ,31^**^ | ,11 | ,14 | ,04 | -,02 | ,07 | ,22 | ,18 |
| 19. AA T1 self-efficacy | 127 | 3,98 | 0,92 |  |  |  |  |  |  |  |  |  |  |  |  |  |  |  |  |  |  |  | ,24^*^ | ,29^**^ | ,10 | ,19 | ,22^*^ | ,14 | ,22^*^ |
| **20. Experienced acceptability, Part II (EA 2), sum** |  | 4,44 | 0,49 | .566 |  |  |  |  |  |  |  |  |  |  |  |  |  |  |  |  |  |  |  | ,65^**^ | ,47^**^ | ,70^**^ | ,77^**^ | ,69^**^ | ,61^**^ |
| 21. EA 2 affective attitude | 102 | 4,59 | 0,55 |  |  |  |  |  |  |  |  |  |  |  |  |  |  |  |  |  |  |  |  |  | ,11 | ,37^**^ | ,46^**^ | ,37^**^ | ,54^**^ |
| 22. EA 2 burden (reversed) | 103 | 4,41 | 1,08 |  |  |  |  |  |  |  |  |  |  |  |  |  |  |  |  |  |  |  |  |  |  | ,07 | -,01 | -,02 | ,00 |
| 23. EA 2 intervention coherence | 102 | 4,63 | 0,61 |  |  |  |  |  |  |  |  |  |  |  |  |  |  |  |  |  |  |  |  |  |  |  | ,60^**^ | ,37^**^ | ,48^**^ |
| 24. EA 2 perceived effectiveness a | 102 | 4,37 | 0,72 |  |  |  |  |  |  |  |  |  |  |  |  |  |  |  |  |  |  |  |  |  |  |  |  | ,62^**^ | ,54^**^ |
| 25. EA 2 perceived effectiveness b | 103 | 4,25 | 0,78 |  |  |  |  |  |  |  |  |  |  |  |  |  |  |  |  |  |  |  |  |  |  |  |  |  | ,58^**^ |
| 26. EA 2 recommendation | 102 | 4,43 | 0,76 |  |  |  |  |  |  |  |  |  |  |  |  |  |  |  |  |  |  |  |  |  |  |  |  |  |  |
| **. Correlation is significant at the 0.01 level (2-tailed). | | | | | | | | | | | | | | | | | | | | | | | | | | | | | |
| *. Correlation is significant at the 0.05 level (2-tailed). | | | | | | | | | | | | | | | | | | | | | | | | | | | | | |

EA 2 affective attitude (26.) and EA 1 affective attitude (7.) not included in the sum variables

If the item 3. EA 1 burden (reversed) deleted α = .739

If the item 16. AA T1 burden (reversed) deleted α = .768

If the item 22. EA 2 burden (reversed) deleted α = .730
