# Supplementary material for: Acceptability, reach and implementation of a training to enhance teachers’ skills in physical activity promotion
Source: BMC Public Health. 2020 Oct 16;20:1568. doi: 10.1186/s12889-020-09653-x (PMC7574409; doi:10.1186/s12889-020-09653-x)
Supplement: Supplementary file 4 — Additional file 4. Anticipated acceptability (AA), Interaction techniques, Part II. [file 12889_2020_9653_MOESM4_ESM.docx]

**Additional file 4. Anticipated acceptability (AA), Interaction techniques, Part II.**

|  | **n** | **Mean** | **SD** | **α** | **1** | **2** | **3** | **4** | **5** | **6** |
| --- | --- | --- | --- | --- | --- | --- | --- | --- | --- | --- |
| **1. Anticipated acceptability, Part II (AA 2), sum** |  | 4.48 | 0.48 | .50 |  | .577^**^ | .628^**^ | .589^**^ | .674^**^ | .613^**^ |
| 2. AA 2 affective attitude | 102 | 4.64 | 0.56 |  |  |  | .352^**^ | -.083 | .354^**^ | .567^**^ |
| 3. AA 2 burden (reversed) | 102 | 4.50 | 0.83 |  |  |  |  | .041 | .232^*^ | .394^**^ |
| 4. AA 2 ethicality (reversed) | 100 | 4.44 | 1.27 |  |  |  |  |  | .196 | -.050 |
| 5. AA 2 perceived effectiveness | 101 | 4.51 | 0.63 |  |  |  |  |  |  | .503^**^ |
| 6. AA 2 self-efficacy | 103 | 4.38 | 0.60 |  |  |  |  |  |  |  |

If the item 4. AA 2 ethicality (reversed) deleted α = .69
