# Supplementary material for: Acceptability, reach and implementation of a training to enhance teachers’ skills in physical activity promotion
Source: BMC Public Health. 2020 Oct 16;20:1568. doi: 10.1186/s12889-020-09653-x (PMC7574409; doi:10.1186/s12889-020-09653-x)
Supplement: Supplementary file 5 — Additional file 5. Types of adaptations and implementations in the teacher workshop delivery by those respondents who report having delivered or intending to deliver the program in some form. [file 12889_2020_9653_MOESM5_ESM.docx]

**Additional file 5. Types of adaptations and implementations in the teacher workshop delivery by those respondents who report having delivered or intending to deliver the program in some form.**

| **Type of adaptation of the teacher workshop** | **Number of reports on this type of adaptation** |
| --- | --- |
| One-time session delivery | 8 |
| Plans for future implementation | 5 |
| Condensed content delivery (not clear if more than one session) | 2 |
| Demo class of the program to colleagues | 1 |
| Workshop 1 delivered | 1 |
| **Type of implementation of the teacher workshop** | **Number of reports on this type of implementation** |
| Spreading the word | 6 |
| Tip sharing to other teachers | 4 |
| Material sharing to other teachers | 3 |
| Having ’walking meetings’ with colleagues | 4 |
| Changes (decreases) in own sedentary behaviour | 3 |
| Mostly only discussions or program presentations | 2 |
| School has acquired new equipment that allows more activity | 2 |
| Using the Learning Café teaching style | 2 |
| Activity breaks in teacher/staff meetings | 2 |
| Arranging activity-promoting events to the entire school | 2 |
| Students are asked to get up and collect materials from teachers’ desk | 2 |
